# Osteoid Osteoma, a Diagnostic Problem: A Series of Atypical and Mimicking Presentations and Review of the Recent Literature

**DOI:** 10.3390/jcm12072721

**Published:** 2023-04-05

**Authors:** Justyna Napora, Szymon Wałejko, Tomasz Mazurek

**Affiliations:** Department of Orthopaedics and Traumatology, Faculty of Medicine, Medical University of Gdańsk, 80-210 Gdańsk, Poland

**Keywords:** osteoid osteoma, benign tumours, benign neoplasms, atypical tumours, thermoablation, intraoperative 3D navigation, disease masking, treatment trends, difficult diagnosis

## Abstract

Osteoid osteoma (OO) is a common benign bone tumour, usually affecting young people. Typically, it is localised to the diaphyses or metaphyses of long bones. The classical manifestation includes distinctive night pain, almost always present, responding well to non-steroidal anti-inflammatory drugs, sometimes accompanied by complaints due to physical activity, and a typical picture on additional tests. A characteristic of osteoid osteoma is the presence of a nidus, usually visible on imaging tests. The nidus generally presents as a single, round lytic lesion up to 1 cm in diameter, surrounded by an area of reactive ossification. However, OO is a multifaceted neoplasm, and its diagnosis can cause numerous difficulties. OO can mimic multiple diseases and vice versa, which often leads to a prolonged diagnostic and therapeutic path and associated complications. There are few literature reviews about the differentiation and diagnostic difficulties of osteoid osteoma. Very effective therapies for this tumour are known, such as ablation and resection. Enhanced detection of osteoid osteoma could result in faster diagnosis and less suffering for the patient, avoidance of complications, and reduced costs of incorrect and prolonged treatment.

## 1. Introduction

Osteoid osteoma (OO) is a common benign neoplasm, accounting for up to 3% of primary bone tumours. It is usually localised to the diaphysis or metaphysis of a long bone, most often of the lower limb [1]. It most frequently affects young people, usually between 5 and 25 years of age. The disease is three times more likely to affect men [2,3].

A typical symptom of osteoid osteoma is night pain, which responds well to non-steroidal anti-inflammatory drugs. Pain usually occurs around the clock, but awakenings from sleep are characteristic, which is particularly troublesome for the patient [4]. The cause of the pain is probably the localised high levels of prostaglandins, especially prostaglandin E, accompanying osteoid osteoma [5,6].

The diagnostic basis is a classical radiograph (X-ray) and then a computed tomography (CT) scan. CT is the method of choice in order to identify a hypodense tumour focus surrounded by a hyperdense sclerotic area [7,8]. Magnetic resonance imaging (MRI) can be helpful in the diagnosis of some cases, presenting with bone marrow oedema and lesions in the adjacent tissues. Dynamic enhanced MRI plays a key role, since nidus enhancement strongly supports diagnosis. Bone scintigraphy, providing a metabolic evaluation of the lesion, can also be helpful in differentiating it from other diseases [9,10]. The typical radiologic sign of osteoid osteoma is a central, X-ray-permeable partially ossifying focus (nidus), surrounded by an area of reactive ossification [6,11]. A combination of clinical features and typical X-ray, CT, and MRI results is usually sufficient to make a reliable diagnosis [12].

Some cases of OO undergo spontaneous regression. However, conservative treatment is rarely administered due to the necessary long-term drug therapy and uncertain clinical outcome. An option is en bloc resection of the lesions; yet for typical OO, the standard treatment is the use of minimally invasive methods. High-frequency electrical current thermoablation (RFA) is considered the gold standard for the treatment of symptomatic cases of this tumour owing to its availability and high efficacy. Treatment with magnetic resonance-guided focused ultrasound (MRgFUS) appears to be a promising alternative as it is non-invasive and comparably efficient; however, it is limited by the strictness of indications and access to MRI [13,14].

Osteoid osteomas with radiological and clinical features other than their classical presentation and located in atypical locations are referred to as atypical [15]. Numerous musculoskeletal conditions may exhibit clinical and radiological features that mimic osteoid osteoma [16]. Osteoid osteoma is also an entity often confused with osteoblastoma, enchondroma, osteochondroma, and other pathological bone lesions [17,18].

Atypically located osteoid osteomas have a higher rate of recurrence and adverse events associated with surgery [19].

Early diagnosis and treatment of the disease can have a significant impact on the quality of life of patients and their families and protect the patient against many unnecessary diagnostic procedures, treatment, and distant complications of the disease [20].

The aim of this report is to discuss the atypical presentation of osteoid osteoma using the example of 11 patients operated on over a nine-year period in the Research Department of Orthopaedics and Traumatology and to review the recent literature on the difficulties of diagnosing osteoid osteoma, the differential diagnoses, and the treatment of choice in these cases.

## 2. Materials and Methods

Ninety-one patients with the diagnosis of osteoid osteoma operated on during a nine-year period (July 2013–July 2022) by thermoablation in the Research Department of Orthopaedics and Traumatology were included in the analysis. Every procedure was performed with the same electrode (BT-1510B, RF Medical Co, Ltd., Seoul, Republic of Korea). Twenty-six (28.6%) women and sixty-five (71.4%) men participated in the study. The mean age of the patients was 20.8 years; 89.9% of the patients initially presented the clinical picture of the disease accepted in the literature as typical. This clinical picture consisted of night pain, usually responding to non-steroidal anti-inflammatory drugs, functional disorders of the affected limb and a typical presentation of osteoid osteoma on diagnostic imaging. Note that 12.1% of the patients had an atypical presentation of the disease, which in most cases resulted in a prolonged and wrong diagnostic path. The lesion was located in the upper limb in 5.5% of the cases and in the lower limb in 94.5% of them. All the patients had an X-ray and CT scan on the diagnostic path done. Following the analysis of the diagnostic path after it ended, the vast majority of cases showed a typical clinical and radiological picture of OO, so RFA was performed. In doubtful cases, a sample was taken for histopathological examination. Sources indicate that the standard biopsy for suspected OO is controversial due to the high variability of histopathological findings [21,22,23]. The patients were also examined using a visual analogue scale (VAS) on the day of the procedure and 2–3 months after the procedure. Their diagnosis was confirmed based on the subsidence of pain after the procedure or the histopathological finding.

## 3. Results

### 3.1. Case 1: OO Masking as Femoroacetabular Impingement (FAI)

**Figure 1:**
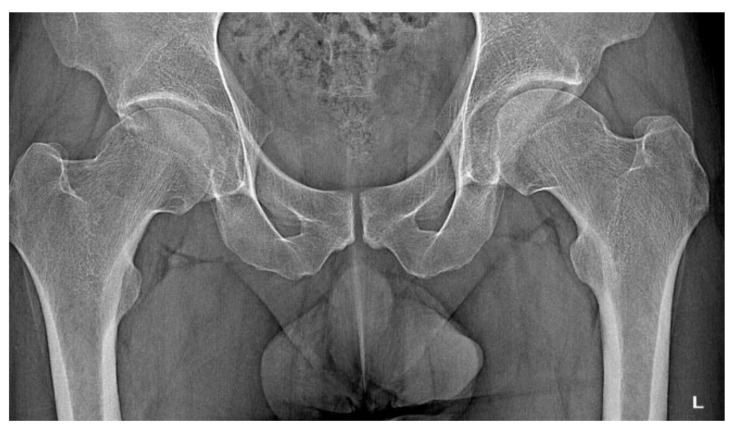
X-ray examination of the hip joints with the features of FAI.

**Figure 2:**
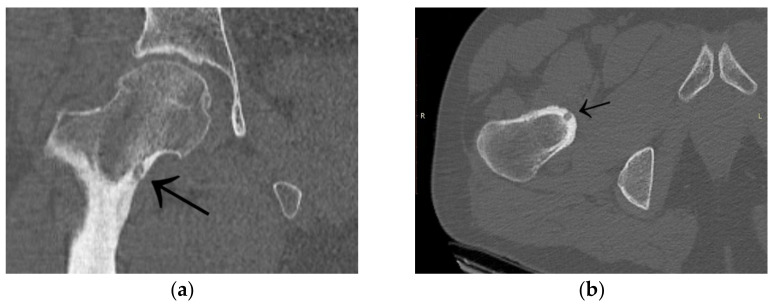
CT scan showing a focus of OO (arrows) in the right femoral neck. (**a**) Frontal CT scan; (**b**) Transverse CT scan.

A 30-year-old man was operated on for OO of the right femoral neck. He had a history of night and post-exercise pain in the right hip for 6 months. On physical examination, positive femoroacetabular impingement-specific tests, FADIR (groin pain on flexion, internal rotation and successive adduction of the hip joint) and FABER (groin pain on flexion, external rotation and abduction of the hip joint) were carried out. After a pelvic X-ray and hip MRI scan, a diagnosis of femoroacetabular impingement was made, and the patient was qualified for arthroscopy (Figure 1). The imaging changes suggested a diagnosis of FAI. While awaiting arthroscopy, an ambiguous clinical picture with a history of night pain prompted the attending physician to perform a CT scan of the hip joints. The scan showed a focus of OO of 9 × 6 × 5 mm in the right femoral neck (Figure 2). The patient was treated with thermoablation with intraoperative 3D navigation at the temperature of 90 degrees for 6 min without complications during and after the procedure. An excellent VAS pain reduction score was achieved from 8 points on the day of surgery to 0 at 3 months after surgery. On physical examination 3 months after surgery, the FADIR and FABER tests were negative. The symptoms of FAI disappeared completely.

### 3.2. Case 2: Example of an Incorrect Therapeutic Path of OO

**Figure 3:**
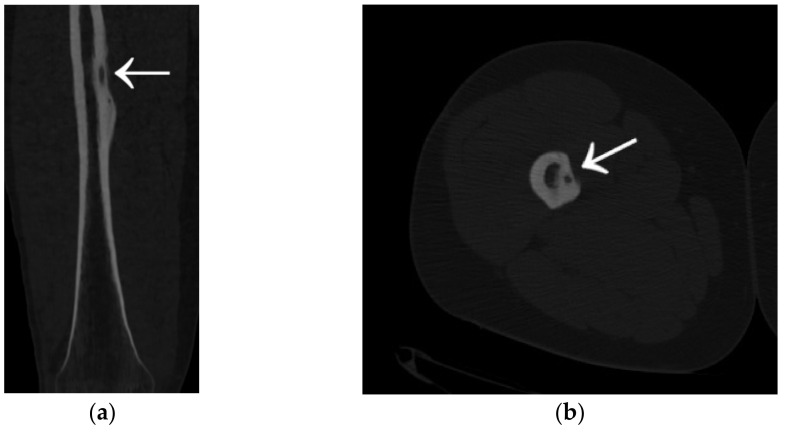
CT scans of the right femur showing flat cortical bone (arrows) after attempted resection and persistent focus of OO. (**a**) Frontal CT scan; (**b**) Transverse CT scan.

**Figure 4:**
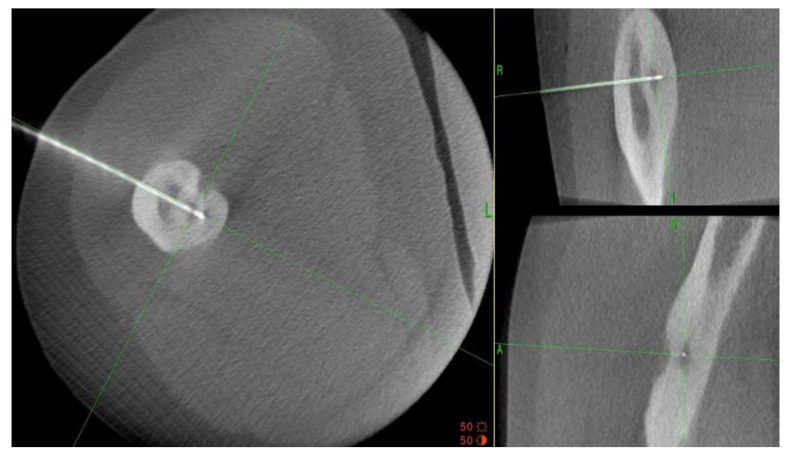
Intraoperative photographs during thermoablation with 3D navigation.

A 35-year-old male patient presented with a diagnosis of inadequately treated osteoid osteoma for two years. The patient informed of pain at night and during the day after exercise or prolonged immobilisation of the limb (sedentary work). The suspicion of OO in the right distal metaphysis of the right femur (a nidus size of 12 × 4 × 4 mm) was raised from the first X-ray examination and a CT scan performed two years earlier. Pain was localised exclusively to the right limb; however, on examination, an incidentaloma was detected in the posterolateral part of the femoral diaphysis on the opposite side with an initial suspicion of a proliferative process. Following an MRI scan of the left knee joint, a suspicion of a non-ossifying fibroma in the healing phase was raised. In a subsequent examination, bone scintigraphy was performed. A focus of pathological tracer accumulation in the right femur was described as a suspected benign lesion, but proliferative disease could not be excluded. After further additional examinations, the patient was qualified for resection of the right femoral tumour. The resection procedure was performed, and the specimen was collected for histopathological examination. After surgery, there was no improvement in terms of pain, and extension contracture of the knee joint (flexion to a level of 120 degrees) developed. The histopathological examination suggested a diagnosis of haemangioma. After further clinical examinations and additional examinations (including a CT scan) (Figure 3), the absence of the features of osteoid osteoma recurrence was described, and pain was associated with the features of a femoropatellar joint injury. Histopathological specimens were re-analysed with the result that there were no features of malignant cells or osteoid osteoma. Over the following months, persistent night pain extending to the anterior surface of the patella and right lower leg was reported. On clinical examination, flexion of the right knee with pain in the medial distal part of the thigh occurred. The subsequent diagnostic process at a rheumatology outpatient clinic suspected gout. The patient was treated with colchicine for several months without any signs of improvement. A second look at the CT scan confirmed a diagnosis of persistent focus of OO. The patient was admitted to the Department of Orthopaedics with muscular atrophy of the right lower limb, impaired mobility (limitation of knee joint flexion of approximately 30 degrees), pain VAS of 6, to perform thermoablation of the lesion resection site. The patient underwent the above procedure under general anaesthesia (Figure 4). On examination the day after surgery, there was a significant reduction in pain (VAS score of 1). On examination 3 months after surgery, there was complete resolution of pain (VAS scope of 0), active flexion of the knee joint without pain, and the patient’s return to activities of daily living. He was recommended to undergo active rehabilitation to restore full limb function.

### 3.3. Case 3: OO Masking as Lyme Disease and Leukaemia

**Figure 5:**
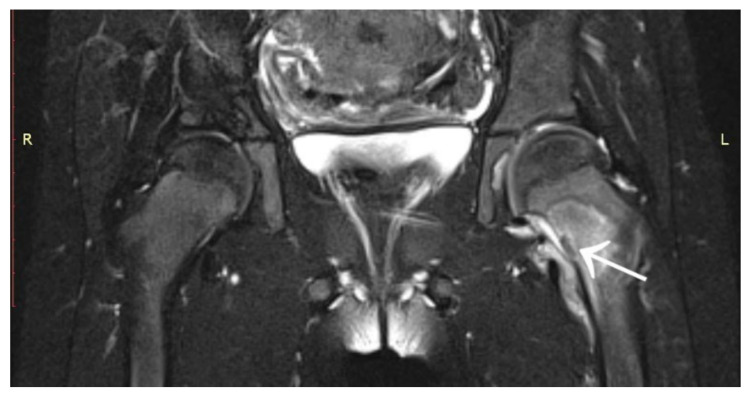
MRI scan showing a focus of OO (arrow) in the left femoral neck.

**Figure 6:**
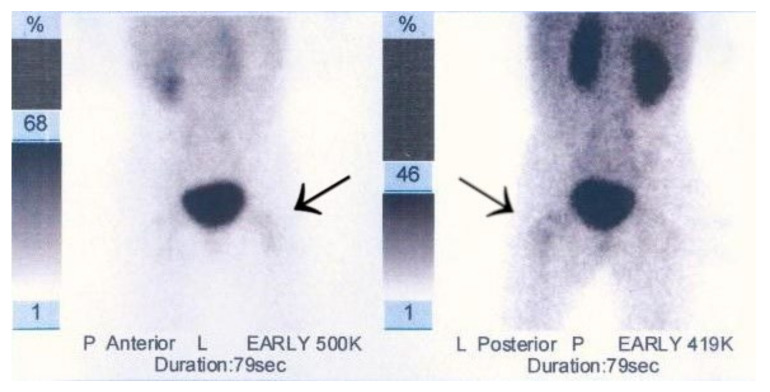
Scintigraphy scan of the patient. Arrows indicate increased radioisotope uptake in the left femoral neck.

**Figure 7:**
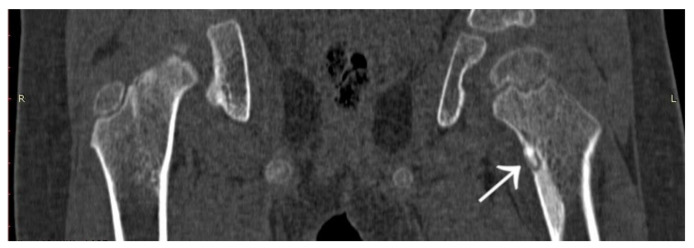
CT scan of the hip joints showing a focus of OO (arrow).

A 6-year-old girl was admitted to the Research Hospital of Orthopaedics for Children and Adolescents with symptoms of night pain (VAS score of 7), especially in the left knee, with lesser intensity in the left hip, accompanied by limping and decreased appetite. The diagnosis made six months earlier detected leucocytosis (23,000), positive IgM antibodies, and Western blot for Lyme disease. A diagnosis of Lyme disease was made, and the patient was treated with antibiotic therapy. The symptoms did not resolve after treatment. The patient underwent an MRI scan of the hip joints (Figure 5). Based on that examination, the patient was diagnosed with a left femoral neck stress lesion with a secondary fracture. The patient also underwent bone scintigraphy, showing a lesion in the left femoral neck (Figure 6). Persistent leucocytosis with the stimulation of granulocytes and features of abnormal cell maturation prompted a double bone marrow trephine biopsy and femoral biopsy. The image of the bone marrow ruled out proliferative disease in all cases. In the meantime, the patient’s emotional state deteriorated with depressive symptoms. The patient was treated by a psychologist with the diagnosis of a psychogenic component accompanying pain. As a result of a further exacerbation of the pain, the patient underwent a CT scan of the hip joints showing a 7 × 5 × 6 mm focus in the left femoral neck with a description corresponding to osteoid osteoma (Figure 7). Thermoablation with intraoperative 3D navigation was performed, resulting in the complete resolution of the symptoms (VAS score of 0 after 2 months).

### 3.4. Case 4: OO Masking as a Fatigue Fracture

**Figure 8:**
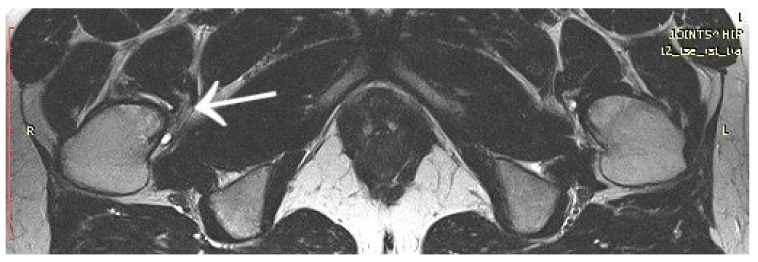
MRI scan of the hip joints; subtle changes (arrow) in the right femoral neck made suspicion of fatigue fracture, no focus of OO revealed initially.

**Figure 9:**
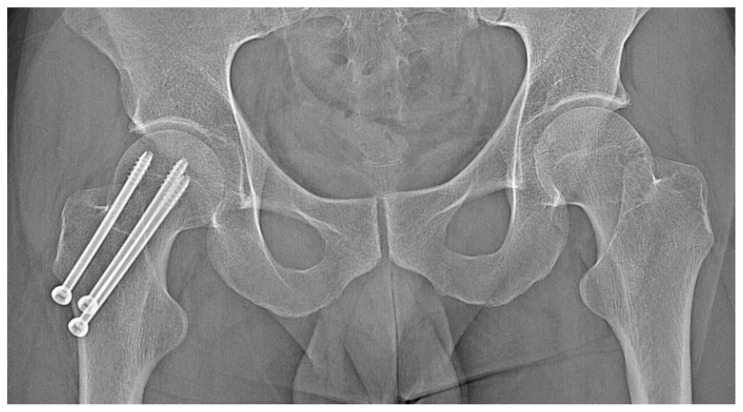
X-ray view of internal stabilisation with three cannulated screws of the right femoral neck.

**Figure 10:**
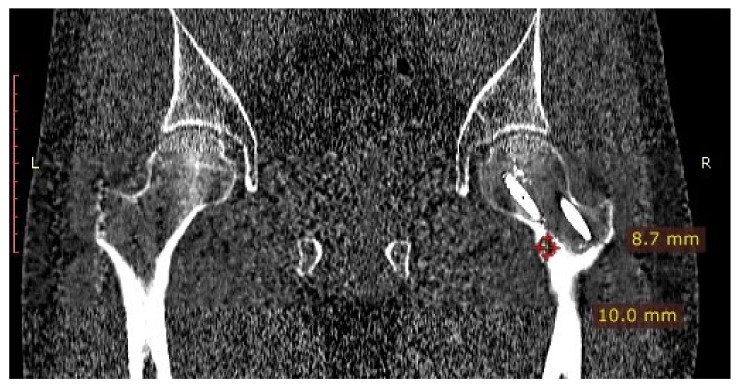
CT scan of the hip joints showing a focus of OO in the right femoral neck.

**Figure 11:**
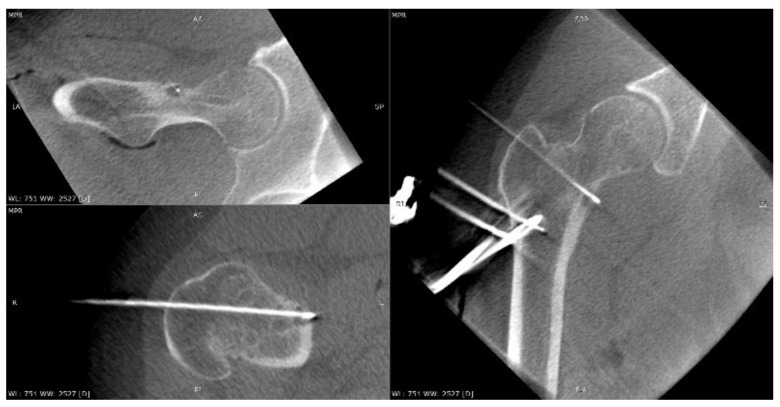
Intraoperative photographs during thermoablation with 3D navigation. Cannulated screws were unscrewed for the time of ablation, and reinserted after the procedure.

A 31-year-old male patient with post-exercise and night pain was seen at an orthopaedic outpatient clinic for diagnosis. The patient underwent a pelvic X-ray and an MRI scan of the right hip joint. The MRI description suggested a suspected fatigue fracture of the right femoral neck (Figure 8). The patient was qualified for surgery. Under general anaesthesia, internal stabilisation was made with three cannulated screws (Figure 9). During the following six months after surgery, the pain did not resolve. During further diagnosis, the patient had a CT scan of the hip joints. The examination revealed a focus of OO of 10 × 9 × 6 mm in the right femoral neck (Figure 10). The patient underwent thermoablation with intraoperative 3D navigation without complications (Figure 11). The VAS score of pain on the day of surgery was 7. Night and postoperative pain resolved completely. The VAS score on examination 3 months after surgery was 0. Cannulated screws were not removed.

### 3.5. Case 5: OO Masking as Tuberculosis and Juvenile Idiopathic Arthritis

The following case is described in more detail in the article [24]. Permission was obtained from the journal editors to use the data, images, and information contained in the article.

**Figure 12:**
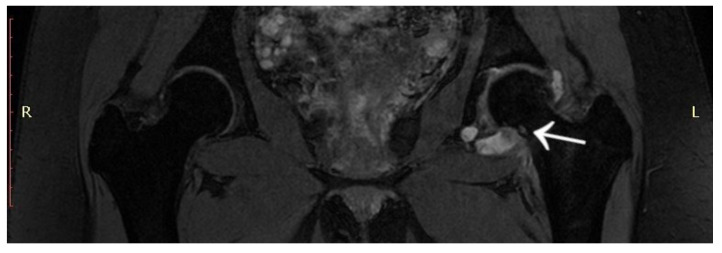
First MRI scan of the hip joints with visible focus of OO (arrow) in the femoral head/neck border, unnoticed.

**Figure 13:**
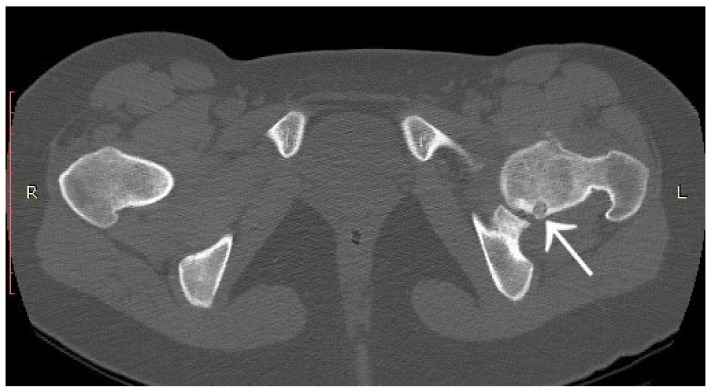
CT scan showing a focus of OO (arrow) in the left femoral head.

A 14-year-old female patient presented with a history of two years of pain in the left hip with a limp in her leg. The pain occurred mainly at night, waking her from her sleep. The girl associated the onset of her symptoms with an injury. The patient gave a history of a prolonged diagnostic process: 12 hospitalisations, four MRI scans, and numerous X-ray and ultrasound examinations. In the results provided, the first MRI scan already showed a small lesion in the left femoral neck, which was not described in the interpretation of the MRI scans (Figure 12). Based on the MRI scan with the description of bone marrow oedema and the features of synovitis, the patient was admitted to the Paediatrics Department with suspected hip arthritis, treated with antibiotic therapy. Six months later, during another hospitalisation for suspected hip arthritis, laboratory tests showed HLA-B27 antigen, weakly positive antinuclear antibodies, and a positive tuberculin test. The patient was urgently hospitalised at the Lung and Tuberculosis Centre with a diagnosis of latent tuberculosis. With suspected tuberculous hip arthritis, a cortical and synovial biopsy was performed. Treatment with isoniazid for 9 months was administered. Synovitis was present in the biopsy specimen. During a subsequent hospitalisation in the Developmental Age Rheumatology Department, juvenile idiopathic arthritis was diagnosed, and HLA-B27 antigen was detected. The patient was started on methotrexate. At subsequent hospitalisations, treatment with chloroquine and sulfasalazine was started, maintaining treatment with methotrexate. With no signs of improvement, the patient was qualified for biological therapy. In the meantime, a suspicion of femoroacetabular impingement was raised during a series of additional examinations. After almost two years of incorrect diagnosis and treatment, a CT scan of the left hip joint was performed for the first time. The image revealed an osteolytic lesion of 7 × 7 × 6 mm (Figure 13). The description showed the features typical of OO. The patient was qualified for thermoablation with intraoperative 3D navigation. On the day of surgery, the patient presented the limitation of mobility of the left hip joint with VAS 8 hip pain. The day after surgery, the VAS score was 3. On examination 2 months after surgery, there was complete resolution of pain with full mobility of the left hip joint.

### 3.6. Case 6: OO Masking as Chronic Hip Arthritis

**Figure 14:**
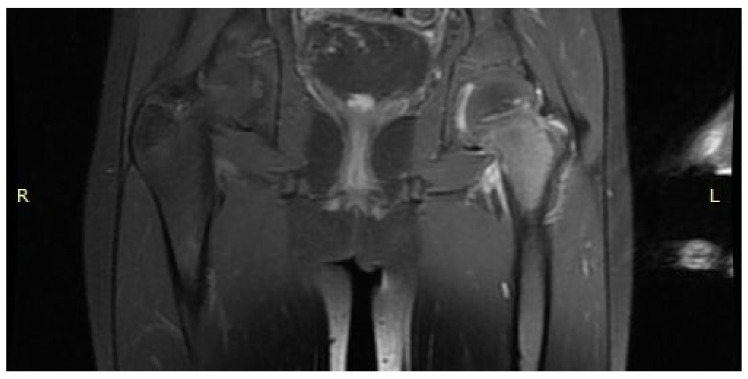
MRI scan of the hip joints showing oedema of the left femoral neck and suggesting inflammation of the hip joint, no nidus revealed.

A ten-year-old female patient had complained of pain for 1.5 years. She had night pain, with no history of injury. The initial orthopaedic examination at the beginning of the diagnostic path showed lower limb asymmetry, muscle atrophy of the left thigh, and the limitation of flexion and external rotation of the left hip joint. On the X-ray, the radiological picture was non-specific. Based on an ultrasound of the hip joints, a diagnosis of a snapping hip was made. Due to oncological concerns, the patient had bone turnover markers and tumour markers performed, all being negative. During further diagnosis of suspected inflammatory and degenerative changes, an MRI scan of the hip joints was performed (Figure 14). The scan description showed inflammatory changes of the left hip joint with changes in the bone marrow of the femoral neck and inflammation of the adjacent tissues. The patient was advised bed rest, treatment with clindamycin, and based on the above examinations, the orthopaedist also suspected a malformation of the lower limb. The complaints of pain persisted. The X-ray examination and MRI scan with the conclusion of a non-specific picture, typical features (bone marrow oedema, neck oedema, and soft tissue reaction), and atypical features (involvement of the metaphysis only) were reconsulted for non-bacterial left femoral osteitis. The patient underwent skeletal scintigraphy showing a lesion in the left femur with no clear features typical of an inflammatory process, for further diagnosis. A bone biopsy was performed with a histopathological result of the absence of a tissue suspicious for a proliferative process. The features of chronic osteitis were found. After the biopsy, another MRI scan was performed with an image of a lesion in the left femoral metaphysis with no change compared to the previous scan.

**Figure 15:**
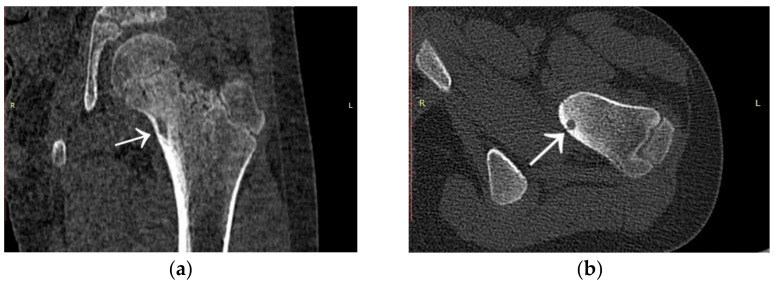
CT scans showing a focus of OO (arrows) in the left femoral neck. (**a**) Frontal CT scan; (**b**) Transverse CT scan.

Due to the worsening of symptoms over the next six months, the patient was admitted to the Children Trauma and Orthopaedic Department for the treatment of arthritis of the left hip and femur. A CT scan was performed, which showed a lesion with a slightly sclerotic edge measuring 10 × 4 × 3 (Figure 15). Based on the previous history, the radiological description considered an abscess as a complication of purulent arthritis. On clinical consultation, a diagnosis of osteoid osteoma, located in the epiphysis of the left femoral neck, was made. The patient underwent transcervical drilling of the focus of osteoid osteoma. No improvement was obtained after drilling. The patient changed the treatment centre, underwent thermoablation with intraoperative 3D navigation, with an immediate healing effect and VAS of 0 at 2 months post treatment. The patient made a full recovery.

### 3.7. Case 7: OO Masking as a Meniscus Injury

**Figure 16:**
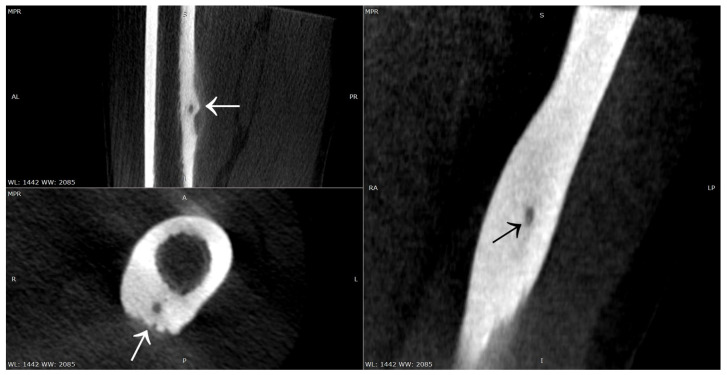
Intraoperative photographs during thermoablation with 3D navigation, showing nidus of OO (arrows) in the femur shaft.

This case involves a 37-year-old man who presented for an orthopaedic consultation due to left knee pain. The pain had occurred for two years, exacerbated after exercise. An MRI scan of the left knee joint was performed, showing damage to the medial meniscus. The patient was qualified for arthroscopy. During surgery, the damaged fragment was removed. Over the following months, the pain in the knee joint worsened. In the diagnostic process, a CT scan of the left knee joint was performed. The examination revealed a focus of OO (6 × 3.5 × 3), located in the distal segment of the left femur. The patient was qualified for thermoablation treatment with intraoperative 3D navigation (Figure 16). On the day of admission, the VAS score was 6. The day after the procedure, the VAS score was 0. The patient is currently undergoing rehabilitation and recovery.

### 3.8. Case 8: OO Masking as Juvenile Idiopathic Arthritis

**Figure 17:**
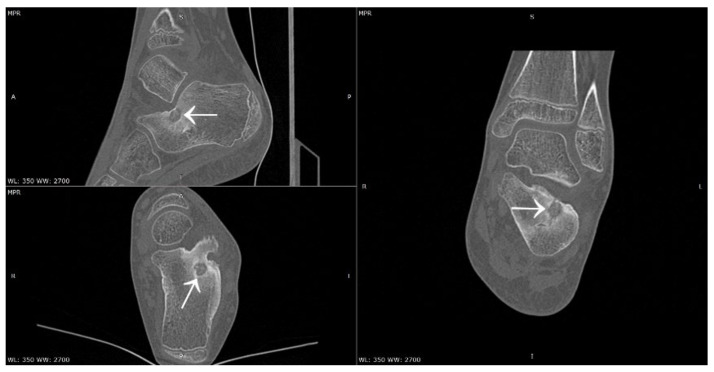
CT scans showing a focus of OO (arrows) in the left calcaneus.

A 9-year-old girl presented to the Department of Orthopaedics for Children and Adolescents with suspected osteoid osteoma and pain, with a VAS score of 7 on the day of admission. The patient followed an extensive rheumatology diagnostic and treatment path. The onset of symptoms occurred 15 months earlier in the form of night pain, oedema, and soreness of the ankle joint and painful limited mobility of the left ankle joint. On ultrasound examination of the ankle joint, lateral malleolus effusion, synovial hypertrophy, and local inflammatory reaction were found. Juvenile idiopathic arthritis was suspected. During the diagnostic process, the infection of Mycoplasma pneumoniae and Borrelia burgdorferi was found, both treated with antibiotic therapy. A high titre of ANA antibodies and the presence of HLA-B27 antigen were detected, and several local injections of glucocorticosteroids were performed. Following further aggravation of symptoms, a diagnosis of juvenile idiopathic arthritis was made, and treatment with sulfasalazine was initiated. Despite the therapy, oedema of the left ankle joint grew. Oral prednisone and methotrexate were added to the therapy. After several months of therapy, oedema of the left ankle joint continued, with increasing pain. The decision was made to qualify the girl for biological treatment. The patient underwent therapy with a biological drug (adalimumab) without any therapeutic effect. During further diagnosis, a CT scan of the left ankle joint was performed, revealing an 8 × 7 × 7 mm focus located in the left calcaneus, pointing to a diagnosis of osteoid osteoma (Figure 17). The patient underwent thermoablation with intraoperative 3D navigation with excellent results (2 months after the procedure, VAS of 0). However, during the months of treatment, the patient’s mental state deteriorated significantly. From her mother’s account, the girl was a cheerful, lively child before treatment. During the treatment process, she became a withdrawn child, with a constant feeling of anxiety. The patient’s mood decreased significantly. The girl made 3 suicide attempts. She remains under psychiatric care.

### 3.9. Other Unusual Cases

**Figure 18:**
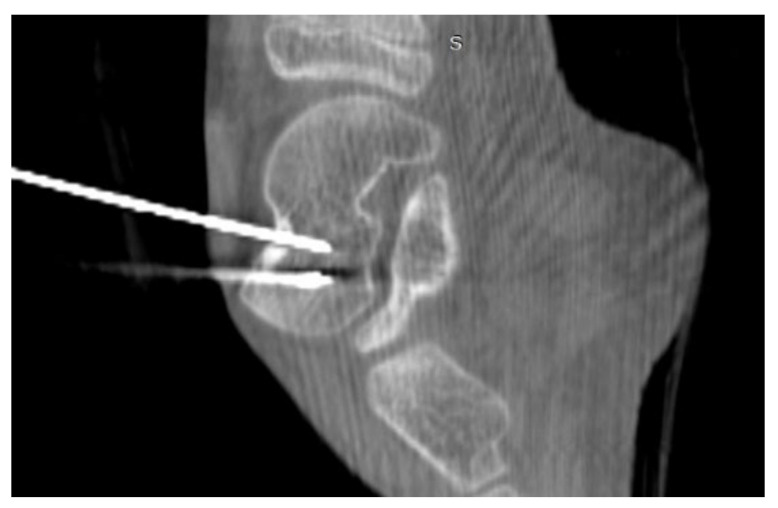
Intraoperative photograph during thermoablation with 3D navigation.

**Figure 19:**
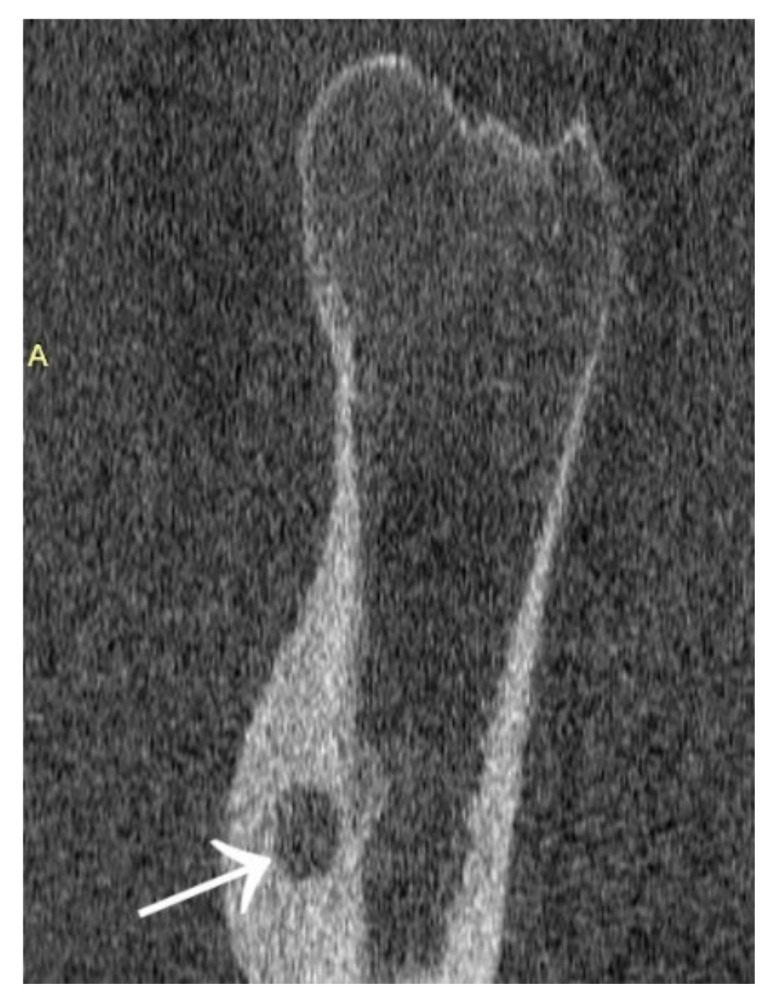
CT scan showing a focus of OO (arrow) in the left lesser trochanter.

**Figure 20:**
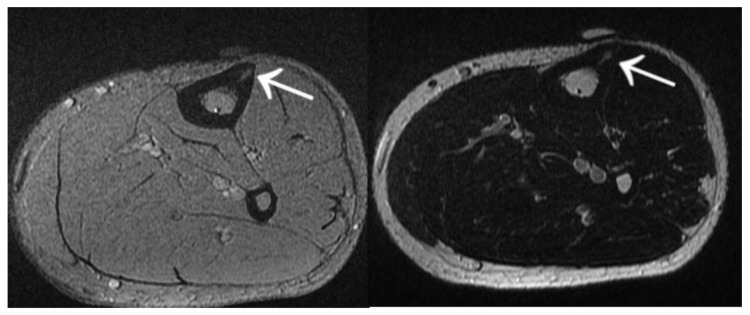
MRI scans showing a focus of OO (arrows) in the left tibia.

Other cases involved a 13-year-old male patient with two foci in the left talus (Figure 18); a 39-year-old man with a focus in the lesser trochanter after left knee arthroscopy due to an image of a meniscus injury as in Case 7 (Figure 19); a 37-year-old male patient with osteoid osteoma of the left tibial diaphysis after surgery for Legg–Calvé–Perthes disease on the same side in childhood, with a prolonged diagnosis focused on complaints related to the past disease (Figure 20).

## 4. Atypical Osteoid Osteomas: Presentation and Diagnosis

The diagnosis of OO is straightforward when there are X-ray-permeable foci surrounded by reactive sclerosis. However, in some cases, diagnostic difficulties may be caused by the atypical appearance or failure of the X-ray to reveal lesions typical of OO and by non-specific location (Table 1) [25,26].

The classical symptom of osteoid osteoma is pain that awakens patients during the night. Other possible symptoms include abnormal bone growth, deformity, painful scoliosis and, if OO is in a joint, also oedema, synovitis, limited joint mobility, and contracture [27,28].

The differential diagnosis of osteoid osteoma includes many conditions, the most common of which are fatigue fracture, chronic osteomyelitis (Brodie’s abscess), cortical desmoid, chondroblastoma, subchondral cyst, enchondroma, intracortical haemangioma, monostotic fibrous dysplasia, healed or healing benign bone cyst, fibrocystic lesion, FAI, neurovascular lesions, and other malignancies [21,29].

### 4.1. Osteoid Osteoma in Unusual Locations

An atypical tumour location can present diagnostic difficulties. Unlike most primary tumours, osteoid osteoma can occur anywhere on the skeleton [21,30]. An uncommon location for osteoid osteoma is the foot, where it usually manifests with uncharacteristic pain and inconclusive radiographs. In a multicentre study described by Smolle, the authors indicated that most osteoid osteomas of the foot were in the metatarsus, followed by the forefoot. Of the patients, 97% had painful lesions [31]. Sources report that the atypical presentation often involves the phalanges, where the main clinical feature is soft tissue oedema and skin tenderness over the tumour [32,33,34,35]. A rare presentation is a painless lesion; however, such a site may be, for example, a subungual location [36]. Sources also include descriptions of the very rare presentation of osteoid osteoma as several foci in a single patient [37,38].

Another unusual presentation is lesions located in the wrist and hand. Among the bones of the wrist, the scaphoid bone is most commonly affected. OO of the carpal region can lead to misdiagnoses, including sterile necrosis of the lunate bone and post-traumatic midcarpal joint synovitis [39,40].

Recent literature also contains descriptions of OO located in the ribs, mandible, sacrum, patella, scapula, as well as atypical locations in long bones such as the olecranon and the capitulum of the humerus [41,42,43,44,45,46].

### 4.2. Mimicking Rheumatologic and Inflammatory Diseases

A common presentation of osteoid osteoma is conditions that mimic rheumatological diseases. Particularly, the periarticular location may lead to misdiagnoses due to mimicking arthritis [47]. In their study, Traore et al. described cases of intraarticular osteoid osteomas in children, incorrectly diagnosed as juvenile idiopathic arthritis, treated with an intraarticular injection of corticosteroids. An accurate diagnosis by a CT scan only enabled the correct choice of treatment [48]. Joint pain that does not respond to conventional treatment requires a persistent diagnosis that takes into account the exclusion of a number of other disorders.

OO can present with severe pain, marked tissue induration, and atypical location, resulting in an incorrect diagnosis of osteomyelitis [49]. Baky et al. presented a case of a female patient with insignificant flow cytometry, in a biopsy, resulting in a diagnosis of chronic recurrent multifocal osteomyelitis (CRMO). The patient was treated with methotrexate, adalimumab, and anti-inflammatory drugs without any success of therapy. A CT scan showed intraarticular OO in the hip, and the use of radiofrequency ablation led to healing [50].

The possibility of a bone tumour in a child with unexplained musculoskeletal pain should always be considered. Osteoid osteoma may co-occur with or clinically mimic musculoskeletal conditions such as chronic shoulder pain [4,51]. Also, fibrous dysplasia and fatigue fractures can cause cortical thickening and proliferation, which can lead to a misdiagnosis of OO [37].

### 4.3. Presentation of Osteoid Osteoma Localised Intraarticularly

Intraarticular osteoid osteoma accounts for approximately 13% of all OOs [52]. It is most commonly located in the hip joint. Significantly less frequently, it is located in the ankle, knee, wrist, and elbow. Pain and joint effusion can cause diagnostic difficulties [29,53]. A lesion localised to the joint can lead to deformity and impaired mobility. Intraarticular OO can often be confused with other causes causing synovitis [54]. It may also resemble intraarticular infection, joint tuberculosis, Legg–Calvé–Perthes disease, and hip impingement syndrome in its presentation [55]. It is particularly difficult to make a diagnosis when the patient has other joint pathology. Histopathological confirmation is not always possible or necessary to make a diagnosis. Histological analyses repeatedly fail to confirm the diagnosis [22,56]. In their study, Song et al. indicated that the characteristic symptom of osteoid osteoma with intraarticular or periarticular location is a painful limitation of joint mobility secondary to synovitis. That symptom was present in 73% of children with OO in the location, but in no child with OO with extraarticular location. The same study found that the classical symptoms of OO such as night pain and NSAID efficacy were present in only 36% of children compared to 70% of patients suffering from OO with extraarticular location [57].

In a study by Dai et al. of arthroscopy of hip osteoid osteoma, the most common reason for revision surgery was the diagnosis of FAI at the first operation. Misdiagnosis as FAI or synovitis is common, especially when OO is localised to the anterior aspect of the femoral head and neck. The clinical presentation of both disease entities is similar and includes pain and limited joint mobility. Computed tomography is the most helpful imaging examination for the diagnosis of intraarticular OO [58]. The delayed diagnosis of osteoid osteoma of the hip can also lead to degenerative changes of this joint with progressive femoral neck hypertrophy and secondary FAI [58,59].

Tomasevich et al. described a case of OO mistaken for a herniated synovial membrane cavity. The patient underwent hip arthroscopy, acetabuloplasty with synovectomy. Further investigations confirmed osteoid osteoma, successfully treated with radiofrequency ablation [60]. A case of osteoid osteoma misdiagnosed as a bone island (enostosis) in a female patient with osteopoikilia was also reported in the literature [61].

### 4.4. Presentation of Osteoid Osteoma Localised in the Axial Skeleton

The lumbar spine is the most common location of spinal OO, which can mimic the painful course of scoliosis. The curvature is caused by asymmetric muscle contraction, and the focus is usually on the concave side of the arch. Spinal osteoid osteoma can also mimic lumbar muscle degeneration and intervertebral disc herniation. OO mimicking intervertebral disc herniation may present with back pain and sciatica as initial symptoms. Tumour resection of this area may provide relief from back pain and symptoms associated with the spinal curvature [62,63].

### 4.5. Similarity to Other Tumors

Histologically similar tumours to osteoid osteoma are osteoblastoma; hence, it is extremely difficult to distinguish between the two conditions. Both tumours occur in young people, usually up to 30 years of age with a male to female sex ratio of 2:1. Osteoblastoma is less likely to present with night pain and usually does not respond to NSAIDs [6,64]. The size of the nidus is used to distinguish osteoid osteoma from osteoblastoma. Osteoblastoma has a nidus usually larger than 1–2 cm. Osteoblastoma, in contrast to OO, has more aggressive local behaviour with often significant bone destruction and a higher rate of recurrence. Osteoblastomas are approximately 4 times rarer than OOs and are more commonly located in the posterior part of the spine [65,66].

There are also numerous case reports of OO mimicking other benign bone tumours (chondroblastoma, glomus tumour, enchondroma, or osteochondroma), which in some cases may be indistinguishable on imaging studies. OO can also mimic malignant bone lesions (metastatic foci, osteosarcoma, or Ewing’s sarcoma). Aggressive lesions often affect the bone marrow and cortical layer, which makes them easier to distinguish [16,17,67,68].

## 5. Diseases Masking as Osteoid Osteoma

Diagnostic difficulties also exist with other diseases that may resemble osteoid osteoma in presentation. Benign bone tumours in the paediatric population are much more common than malignant ones. They may have a unique presentation that helps narrow down the diagnosis; however, there are cases which pose differential diagnostic difficulty [51].

### 5.1. Benign Tumors Resembling Osteoid Osteoma

Ramnath et al. described two cases of intracortical chondroma mimicking osteoid osteoma. Both cases underwent radiofrequency ablation, one of which resulted in the healing of intracortical chondroma and the other was unsuccessful and required further surgical treatment [69]. Cao et al. described a case of enchondroma of the proximal femoral epiphysis, misdiagnosed as OO, treated with resection [70]. Chondroblastoma in epiphyseal locations in children may resemble osteoid osteoma; however, the epiphyseal location and intramedullary position are more characteristic of chondroblastoma, whereas OOs are usually diaphyseal and intracortical [37]. Intramuscular haemangioma, occurring close to a bone, can result in cortical, medullary, and periosteal lesions, which are often misdiagnosed on a classical X-ray. Misinterpretation may be due to their rarity, deep location, or unusual presentation of the lesion [71].

### 5.2. Other Conditions Mimicking Osteoid Osteoma

Enthesopathies presenting with atypical periosteal reactions can mimic primary and secondary bone tumours. Tendon enthesopathy may mimic osteoid osteoma and should be considered in the differential diagnosis [3].

Brodie’s abscess is repeatedly a diagnostic dilemma that clinically mimics malignant and benign lesions, including OO. The radiological picture of Brodie’s abscess may also suggest the diagnosis of osteoid osteoma. The presence of a sinus tract on computed tomography is almost diagnostic for Brodie’s abscess; hence, a CT scan should be the examination of choice in differentiating these disease entities [72,73].

## 6. Treatment of Atypical Osteoid Osteomas

Notwithstanding the difficulty and prolonged diagnostic process, the treatment of atypical osteoid osteomas does not differ significantly from that of typically presenting OOs.

The most preferred treatment options include resection of the nidus with minimally invasive intraoperative curettage and percutaneous radiofrequency thermoablation (PRT) of the lesion under computerised tomography (CT) guidance [74,75].

Jackle et al. presented findings suggesting that thermoablation can be used as a minimally invasive method to eliminate OO localised to different areas of the epiphyseal cartilage. A study in lambs indicated only localised tissue damage [76].

For tumours that cannot be treated by thermoablation, en bloc resection is the technique of choice. Resection is also the optimal treatment for most spinal OOs [77].

However, in cases of OO localised to a hand, en bloc resection often requires bone grafting. Unroofing and curettage with the burr-down method seems to be effective in preventing residual tumours or relapses of OO of the hand [78].

Also, the use of ablation is restricted by the availability of specialised equipment and qualified staff. An alternative to this method described in the literature for centres lacking modern equipment is CT-guided Kirschner wire placement in a radiology department, and then a surgical intervention in an operating room without guiding Kirschner wire removal [79].

Minimally invasive treatment has some limitations, also depending on the ablation technique used. The main exclusion criteria are proximity to neurovascular structures, poor soft tissue coverage, or location in small bones or around articular cartilage [65,80,81].

The literature contains case reports in which intraarticular osteoid osteoma was resected by arthroscopic methods with excellent postoperative results. Indications for arthroscopy especially include locations that are both intraarticular and difficult to access for ablation techniques [82,83,84].

Notwithstanding the difference in presentation of OO and osteoblastoma, the treatment methods of choice for both tumours are ablation techniques. For lesions not qualifying for treatment by ablation, the technique of choice is resection of the lesion. Therefore, this does not change the management, even when it is difficult to distinguish between the above disease entities [65].

## 7. Conclusions

Despite improvements in imaging techniques, the diagnosis of OO in an unusual location or presenting with atypical symptoms remains a challenge. Data obtained from the patient’s history, clinical examination, and diagnostic imaging are fundamental to making the correct diagnosis. Delaying the diagnosis may lead to patients’ prolonged suffering, reduced quality of life, increased costs associated with inappropriate treatment, and secondary complications caused by some OOs. Furthermore, improved awareness of atypical clinical pictures and symptoms of osteoid osteoma should accelerate diagnosis and referral for appropriate treatment. CT scanning is the method of choice when osteoid osteoma is suspected or differentiated and should be performed in the diagnostic process when the distinction is made between OO and other conditions. En bloc resection and ablation techniques with intraoperative 3D navigation are the methods of choice for the treatment of both typically and atypically presenting osteoid osteomas.

### Limitations

Only patients undergoing thermoablation were included in the study. Patients treated by en bloc resection or other open resection methods were not included due to the ambiguity of diagnoses and repeatedly non-diagnostic biopsies.

## Figures and Tables

**Table 1:** Comparison of typical and atypical features of osteoid osteoma [25,26,27,28].

Feature of OO	Typical	Atypical
Type of bone	Long	Short, irregular
Bone location	Diaphyseal; metaphyseal	Epiphyseal; intraarticular
Most commonly affected bones	Femur, tibia, humerus	Other
Nidus quantity	1	>1
Nidus size	<10 mm	>10 mm
Nidus location	Cortical, subcortical	Intramedullary
Age of patient	<30 yrs.	>30 yrs.
Occurrence of pain	Night pain, awakening, rarely post-exercise	Day pain
Characteristics of pain	Responding to NSAIDs	Non-responding to NSAIDs

## Data Availability

Not applicable.
